# Management of Schneiderian membrane perforations during maxillary sinus floor augmentation with lateral approach in relation to subsequent implant survival rates: a systematic review and meta-analysis

**DOI:** 10.1186/s40729-021-00346-7

**Published:** 2021-07-12

**Authors:** Luis Alfredo Díaz-Olivares, Jorge Cortés-Bretón Brinkmann, Natalia Martínez-Rodríguez, José María Martínez-González, Juan López-Quiles, Isabel Leco-Berrocal, Cristina Meniz-García

**Affiliations:** Department of Dental Clinical Specialties, Faculty of Dentistry, Complutense University of Madrid, Pza Ramon y Cajal s/n, 28040 Madrid, Spain

**Keywords:** Alveolar ridge atrophy, Bone augmentation, Lateral approach, Maxillary sinus floor elevation, Systematic review

## Abstract

**Background:**

This systematic review aimed to propose a treatment protocol for repairing intraoperative perforation of the Schneiderian membrane during maxillary sinus floor augmentation (MSFA) procedures with lateral window technique. In turn, to assess subsequent implant survival rates placed below repaired membranes compared with intact membranes and therefore determine whether membrane perforation constitutes a risk factor for implant survival.

**Material and methods:**

This review was conducted according to PRISMA guidelines. Two independent reviewers conducted an electronic search for articles published between 2008 and April 30, 2020, in four databases: (1) The National Library of Medicine (MEDLINE/PubMed) via Ovid; (2) Web of Science (WOS); (3) SCOPUS; and (4) Cochrane Central Register of Controlled Trials (CENTRAL); also, a complementary handsearch was carried out. The Newcastle-Ottawa Quality Assessment Scale was used to assess the quality of evidence in the studies reviewed.

**Results:**

Seven articles fulfilled the inclusion criteria and were analyzed. A total of 1598 sinus lift surgeries were included, allowing the placement of 3604 implants. A total of 1115 implants were placed under previously perforated and repaired membranes, obtaining a survival rate of 97.68%, while 2495 implants were placed below sinus membranes that were not damaged during surgery, obtaining a survival rate of 98.88%. The rate of Schneiderian membrane perforation shown in the systematic review was 30.6%. In the articles reviewed, the most widely used technique for repairing perforated membranes was collagen membrane repair.

**Conclusions:**

Schneiderian membrane perforation during MFSA procedures with lateral approach is not a risk factor for dental implant survival (*p*=0.229; RR 0.977; 95% CI 0.941-1.015). The knowledge of the exact size of the membrane perforation is essential for deciding on the right treatment plan.

**Supplementary Information:**

The online version contains supplementary material available at 10.1186/s40729-021-00346-7.

## Introduction

Maxillary sinus floor augmentation (MSFA) surgery with simultaneous or deferred placement of implants is a highly predictable surgical technique in cases of atrophic posterior maxilla [1], allowing implant-supported rehabilitation in cases where there would otherwise be insufficient remaining bone substrate for implant placement. MSFA achieves implant survival rates similar to those achieved in pristine bone [2, 3]. During this surgical procedure, perforations of the sinus membrane may occur due either to iatrogenic causes derived from incorrect surgical handling or to anatomical considerations inherent to the individual patient, which can make the procedure difficult [4, 5]. The former include uncontrolled pressure on the membrane or the use of inappropriate surgical instruments [6]; the latter include reduced thickness of the membrane [7], reduced friability and elasticity [8, 9], greater adhesion to the bone surface [10], and the presence of the sinus septa [11–13].

Membrane perforation is the most frequent complication in this type of procedure [14]. According to the various authors reviewed, percentages vary between 7 and 60% [12, 15–18].

In most cases, this complication is corrected intra-operatively [19]. However, the treatments described to repair these perforations are diverse [20].

To our knowledge, no systematic review has attempted to propose specific treatment guidelines in relation to perforation size. Therefore, this systematic review aimed to answer following statement of questions: What is the rate of Schneiderian membrane perforation during MSFA procedures? What is the survival rate of dental implants placed in perforated membranes once they have been repaired compared with the survival rate in intact membranes? How are these perforations treated (depending on their extent)? What are the most frequently occurring complications following membrane perforation?

## Material and methods

### Databases and search strategy

This systematic review was designed to fulfill PRISMA [21] (Preferred Reporting Items for Systematic Review and Meta-Analyses) guidelines and answer the following focused PECO question: “How are Schneiderian membrane perforations that occur during maxillary sinus floor augmentation (MSFA) with lateral approach managed, and does perforation influence subsequent implant survival rates?”

(P) Patient/population: Adult human patients requiring maxillary sinus floor augmentation with lateral approach for subsequent placement of dental implants.

(E) Exposure: Repairing sinus membrane perforations during maxillary sinus floor augmentation.

(C) Comparison: Maxillary sinus floor augmentation without membrane perforation.

(O) Outcomes: Schneiderian membrane perforation rate during MSFA with lateral approach; available therapeutic procedures for repairing these perforations; associated complications of Schneiderian membrane perforations; survival rates of implants in perforated vs. non-perforated membranes.

An electronic search was conducted in four electronic databases: (1) The National Library of Medicine (MEDLINE/Pubmed) via Ovid; (2) Web of Science (WOS); (3) Cochrane Central Register of Controlled Trials (CENTRAL); and (4) Scopus. The search included studies published in English, Spanish, and German published between 2008 and April 30, 2020. The electronic search was complemented by a manual search in Oral and Maxillofacial Surgery and Implant Dentistry related journals and in the reference sections of the studies reviewed. To perform the screening process, all the references were included into EndNote X9 Library (Clarivate Analytics, Philadelphia, PE, USA).

The search used the following key terms combined with Boolean operators (Table 1): (maxillary sinus floor augmentation OR dental implant) AND (sinus lift surgery OR membrane perforation) AND (management of Schneiderian membrane perforations) AND (sinus membrane perforation OR dental implant) AND (maxillary sinus membrane repair) AND (Schneiderian membrane repair OR maxillary sinus floor augmentation) AND (repair system for sinus membrane perforations).

**Table 1:** Information about search strategy, based on PECO question and MeSH index terms, Boolean terms, and its truncations

**Focused question (PECO)**	How are Schneiderian membrane perforations that occur during maxillary sinus floor augmentation (MSFA) with lateral approach managed, and does perforation influence subsequent implant survival rates?	
**P (Population)**	Adult human patients requiring maxillary sinus floor augmentation with lateral approach for subsequent placement of dental implants.	1# (maxillary sinus floor augmentation OR membrane perforation) AND (dental implant)
**E (Exposure)**	Repairing sinus membrane perforations during maxillary sinus floor augmentation.	2# (sinus lift surgery OR membrane perforation OR management of Schneiderian membrane perforations OR repair system for sinus membrane perforations) AND (dental implant)
**C (Comparison)**	Maxillary sinus floor augmentation without membrane perforation.	3# (maxillary sinus floor augmentation OR sinus lift surgery) AND (dental implant)
**O (Outcome)**	Schneiderian membrane perforation rate during MSFA with lateral approach; available therapeutic procedures for repairing these perforations; associated complications of Schneiderian membrane perforations; survival rates of implants in perforated vs. non-perforated membranes.	4# (implant survival rate OR complication OR outcome)
**Search combination Pubmed, Web of Science, Cochrane library, and Scopus**	1# AND 2# AND 3# AND 4#	
**Terms truncation Pubmed, Web of Science, Cochrane library, and Scopus**	(maxillary sinus floor augmentation OR dental implant) AND (sinus lift surgery OR membrane perforation) AND (management of Schneiderian membrane perforations) AND (sinus membrane perforation OR dental implant) AND (maxillary sinus membrane repair) AND (Schneiderian membrane repair OR maxillary sinus floor augmentation) AND (repair system for sinus membrane perforations) AND (implant survival rate OR complication OR outcome)	

Two reviewers (LADO and JC-BB) conducted the primary selection of the articles identified in the electronic and manual search by independently screening the titles and abstracts. The same reviewers selected the full manuscripts of studies that met the inclusion criteria, or those with insufficient data in the title and abstract to reach a clear decision. Any disagreement between the reviewers was resolved by discussion with a third reviewer (CMG). Inter-reviewer reliability (percentage of agreement and kappa correlation coefficient) in full-text analysis was calculated.

The same two reviewers performed data extraction independently in duplicate. Where data were incomplete or missing from a study, the authors were contacted for clarification. When the results of a study were published more than once, only the longest follow-up was included.

### Inclusion and exclusion of studies

The following inclusion criteria were applied:

1. Clinical studies reporting: number of patients included, number of sinus lifts performed, number of perforations produced during sinus lifts, dental implant survival rates.
2. Randomized controlled clinical trials, cohort studies, and case-control studies.
3. Human studies with sample sizes greater than 15.
4. MSFA procedures using the lateral window technique.
5. Studies involving both perforated and non-perforated membranes.
6. Studies reporting the therapeutic options adopted to resolve the membrane perforation.
7. Follow-up of at least 6 month.
8. Articles published in English, German, or Spanish.

The following criteria lead to exclusion:

1. Non-human studies.
2. Articles not in English, German, or Spanish.
3. Studies for which the full text was not available.
4. Case reports.

### Data extraction

Data from each included article was collected by the reviewers (LADO and JC-BB) working together and recorded in an Excel sheet (Version 15.17, Microsoft Inc. 2015), including the following parameters: authors, year of publication, study design, number of patients, number of MSFA, occurrence of membrane perforation during MSFA procedures applying the lateral window technique, perforation rate, perforation size, number of implants, the subsequent survival rates of implants placed below perforated vs. non-perforated membranes, treatments used to repair sinus perforations, and any additional complications produced in sinus lift surgeries with repaired membranes.

### Risk of bias assessment within the studies

The Newcastle-Ottawa scale (NOS) for cohort studies [22] was used to assess risk of bias in individual observational studies and non-randomized trials adapted by Moraschini et al. [23] in 2017. This scale included a questionnaire divided into 3 categories: selection (4 questions), comparability (2 question), and exposure (3 questions). The resulting score can reach a maximum of nine points. Studies were classified as good, fair, or poor-quality (GQ, FQ, or PQ) following the score algorithm proposed by the Agency for Healthcare Research and Quality [24].

### Statistical analysis

Data describing Schneiderian membrane perforation management, perforation rate, and complications associated with perforation were entered on a spreadsheet for data analysis. The relationship between perforation size and implant survival was also analyzed.

Statistical analysis was performed using the STATA© program (Version 15). The results for the survival rate of implants inserted below perforated membranes compared to the survival rate of implants inserted below non-perforated membranes were compared by means of meta-analysis assuming a random effects model. Data were entered on a spreadsheet to perform the meta-analysis with two outcome categories: perforated vs. non-perforated membranes. The measure of effect used was the risk ratio (RR) or relative risk which constitutes the relative measure of effect by indicating how much more often the event tended to develop in the group of subjects exposed (perforated membrane) to the exposure factor or risk factor in relation to the non-exposed group (non-perforated membrane).

Heterogeneity was evaluated using the chi^2^ test and the *I*^2^ statistic. The significance level was set at *p* 0.05 and 95% confidence interval (CI).

## Results

### Screening process

**Fig. 1:**
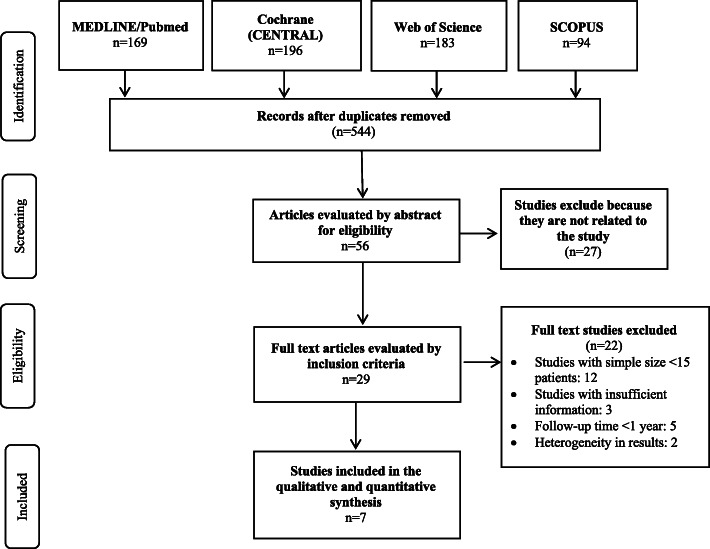
PRISMA flowchart containing the search strategy and the respective selection process

The initial electronic search for the management of Schneiderian membrane perforation during MSFA procedures applying the lateral window technique located 642 articles. Duplicates were discarded and the titles and abstracts screened applying inclusion and exclusion criteria, leaving a total of 56 articles. Of these, 27 articles were excluded because they were in vitro, ex vivo studies, or case reports or because their content was irrelevant to the present review’s objectives (Table 2). A total of 29 articles were selected for full reading. After a thorough analysis, 22 articles were excluded and so seven studies were included in qualitative and quantitative synthesis. A flow chart (Fig. 1) illustrates the entire search and selection process.

**Table 2:** Articles excluded and reasons for exclusion

Reason for exclusion	Study
**Ex vivo study/experimental study**	Yanfeng Li et al. [25], Zhai et al. [9], Alsabbagh et al. [26].
**A case report**	Xiaojun Ding et al. [27], Huang et al. [28], Testori et al. [29], Taschieri et al. [30], Pikos . [31], Meleo et al. [32], Gehrke et al. [33], Fathima et al. [34], Bassi et al. [35].
**No report implant survival rate under membrane perforation vs. non perforation**	Dragonas et al. [36], Sakkas et al. [37], De Oliveira H et al. [38], Shiffler et al. [17], Chirilă et al. [39], Barbu et al. [5], Starch-Jensen et al. [3], Riben et al. [40].
**Only patients with perforated membranes were included**	Kim et al. [41], Nooh et al. [42].
**No lateral approach (solely)**	Chaushu et al. [43], Yoko Oba et al. [44], Giudice et al. [45], Attar et al. [46], Becker ST et al. [12].

### Study characteristics

The seven articles selected for qualitative and quantitative synthesis were retrospective cohort studies published between 2008 and 2020. Table 3 summarizes the information extracted from each study: authors, year of publication, type of study, number of patients treated, number of MSFA with lateral approach performed, number of perforations produced during surgery, percentage of perforations recorded, management of the perforation performed, and the main complications found in cases in which Schneiderian membrane perforation occurred.

**Table 3:** Information about selected studies including number of patients treated, number of MSFA performed, number of perforations, percentage of perforations recorded, management of the perforation performed, and main complications

Author/year	Type of study	Patients (number)	Sinus lift (number)	Perforations (number)	Perforations rate	Management of perforations	Complications
**Park et al.** [4]	Retrospective cohort	63	65	24	39%	Clot formation	Infection
**Beck-Broichsitter et al.** [47]	Retrospective cohort	63	79	39	49.3%	<5 mm: collagen memb or fibrin glue or clot. >5 mm: suturing + collagen memb	Periimplantitis
**Ferreira et al.** [16]	Retrospective cohort	531	745	237	31.8%	Collagen membr + Reabsorbable suture for all perforations	Areas with chronic inflammatory infiltration
**Öncü E et al.** [19]	Retrospective cohort	16	20	10	50%	PRF (<10 mm 햰)	
**Froum et al.** [48]	Retrospective cohort	23	40	15	37.5%	Resorbable collagen membrane (<10 mm 햰)	
**Oh E et al.** [49]	Retrospective cohort	128	175	60	34%	Resorbable hemostatic agente Surgicel© (small-moderate perforations)	Infection in 3 of the 60 perforations
**Hernández-Alfaro et al.** [50]	Retrospective cohort	338	474	104	22%	0-5햰-collagen membr o suturing 5-10햰-collagen membr + laminar bone >10 햰-laminar bone, buccal fat pad, mandibular bone block	Pain/sensitivity
**Total**		**1162**	**1598**	**489**	**30.6%**	**The main treatment was collagen membrane**	**Infection was the most frequent complication**

Table 4 details the total number of implants placed in each study, the number of implants placed below perforated membranes, the number of implants placed below non-perforated membranes, the survival rates (%) of implants placed under intact and perforated membranes and main follow-up of each study.

**Table 4:** Information about total number of implants placed, number of implants placed below perforated membranes, number of implants placed below non-perforated membranes and their survival rates (%), and mean follow-up period of each study

Author/year	Implants (number)	Implants inserted under perforated membranes (number)	Implants inserted under intact membranes (number)	Implant survival rate in perforated membranes	Implant survival rate in intact membranes	Mean follow-up (months)
**Park et al.** [4]	122	44	78	100%	100%	Perforation group: 11.52 (±6.6) Control group: 10.38 (6.73)
**Beck-Broichsitter et al.** [47]	175	92	89	98.9%	100%	Perforation group: 31 (±24) Control group: 20 (±18)
**Ferreira et al.** [16]	1588	523	1065	97.1%	97.7%	Perforation group: 24 Control group: 24
**Öncü E et al.** [19]	35	15	20	100%	100%	Perforation group: 6-12 Control group: 6-12
**Froum et al.** [48]	80	35	45	100%	95.5%	Perforation group:6-32 Control group: 6-32
**Oh E et al.** [49]	438	134	304	97.01%	99%	6-32
**Hernández-Alfaro et al.** [50]	1166	272	894	90.81%	100%	12
***Total***	**3604**	**1115**	**2495**	**97.68%**	**98.88%**	

Table 5 shows the results of qualitative and quantitative synthesis of the studies analyzed. It was found that as the size of the Schneiderian membrane perforations increased so did the failure rate of the implants placed below perforated membranes. Only one study, Beck-Broichsitter et al., did not report the size of the perforations adjacent to failed implants [47].

**Table 5:** Correlation between the size of the Schneiderian membrane perforations and the failure rate of the implants placed below perforated membranes

Author/year	Perforation size (in mm)	Implant failure rate (in perforated membranes)	Management of Schneiderian membrane perforations
**Park et al.** [4]	<5 5-10 >10	0% (0 de 44) (not specified where each implant was inserted)	Clot formation
**Beck-Broichsitter et al.** [47]	<5 >5	1,09% (1 de 92) (not specified the size of the perforations where the implant fails)	<5 mm: collagen membrane or fibrin glue or clot. >5 mm: suturing + collagen membrane
**Ferreira et al.** [16]	<5 5-10 >10	2.3% (6 de 266) 2.7% (4 de 150) 4.7% (5 de 107)	Collagen membrane + reabsorbable suture for all perforations
**Öncü E et al.** [19]	<10	0% (0 de 15)	PRF (<10 mm ⍉)
**Froum et al.** [48]	<10	0% (0 de 35)	Resorbable collagen membrane (<10 mm ⍉)
**Oh E et al.** [49]	5-10	3% (4 de 134)	Resorbable hemostatic agente Surgicel© (small-moderate perforations)
**Hernández-Alfaro et al.** [50]	<5 5-10 >10	2,86% (4 de 140) 8,11% (6 de 74) 25,14% (15 de 58)	0-5⍉—Collagen membrane or (please add "r") suturing 5-10⍉—Collagen membrane + laminar bone >10 ⍉—Laminar bone, buccal fat pad, mandibular bone block

### Patient characteristics

A total of 1162 patients, with an average age of 56 years undergoing 1598 MSFA procedures with lateral window approach, were recruited in the seven studies. The mean Schneiderian membrane perforation rate was 30.6% (489 perforations). Different treatments were used to resolve the perforations, including post-perforation clot formation [4], suturing [47], use of collagen membranes [16], Platelet-rich fibrin (PRF) [19], hemostatic agents [49], laminar bone [50], and block grafts [50].

Of the treatments carried out, it was observed that collagen membrane was the most commonly used material for repairing membrane perforation, regardless of its size. This material was used in four of the seven articles reviewed.

The most common post-operative complication found in sinuses with perforated membranes was the appearance of signs of infection. Park et al. [4] reported a greater number of postoperative complications in patients with perforated membranes including bleeding from the perforation site, leakage of cystic fluid or purulent exudate, displacement of the graft into the sinus, nasal bleeding, and facial swelling. Other complications with lower incidence such as pain or tenderness in the treated area were also reported [4].

A total of 1598 MSFAs with lateral window technique were performed followed by the placement of 3604 dental implants. A total of 1115 implants were placed beneath previously perforated and repaired membranes, obtaining a survival rate of 97.68%, while 2495 implants were placed under intact sinus membranes obtaining a survival rate of 98.88%.

The survival criteria for dental implants in the seven articles reviewed were as follows: loaded implants, which remained in situ, without presenting mobility, free of radiotranslucency and peri-implant infection, and without associated pain (whether spontaneous or under pressure).

After loading the implants, the mean follow-up time of the patients (1162) in the seven studies ranged from 6 to 32 months.

### Inter-reviewer agreement

The inter-reviewer Kappa statistic between the two reviewers (LADO and JC-BB) was 0.856±0.072 (CI 95%, 0.716-0.997).

The intervention of a third reviewer for consensus purposes was not needed.

### Risk of bias

The Newcastle-Ottawa scale [22] allowed to classify the studies included in the systematic review as follows: 2 studies [48, 50] scored 7 points and 3 studies [4, 47, 49] scored 6 points. This indicates a low risk of bias and high methodological quality. Only one study [19] scored 5 points (Table 6).

**Table 6:** Quality assessment of included studies using the Newcastle-Ottawa scale

	Park et al. [4]	Beck-Broichsitter et al. [47]	Ferreira et al. [16]	Öncü E et al. [19]	Froum et al. [48]	Oh E et al. [49]	Hernández-Alfaro et al. [50]
**Selection**							
• Representativeness of the exposed cohort	0	0	0	0	*	0	*
• Selection of the non-exposed cohort	*	*	*	*	*	*	*
• Ascertainment of exposure	*	*	*	*	*	*	*
• Demonstration that outcome of interest was not present at start of study	*	*	*	*	*	*	*
**Comparability**							
• Study controls for bone ring group	0	0	0	0	0	0	0
• Study controls for any additional factor (duration of exposure)	*	*	*	*	*	*	*
**Outcome**							
• Assessment of outcome	0	0	0	0	0	0	*
• Was follow-up long enough for outcomes to occur?	*	*	*	0	*	*	0
• Adequacy of follow-up of cohorts	*	*	*	*	*	*	*
**Newcastle-Ottawa scale**	6	6	6	5	7	6	7

0 no

*Yes

### Meta-analysis

**Fig. 2:**
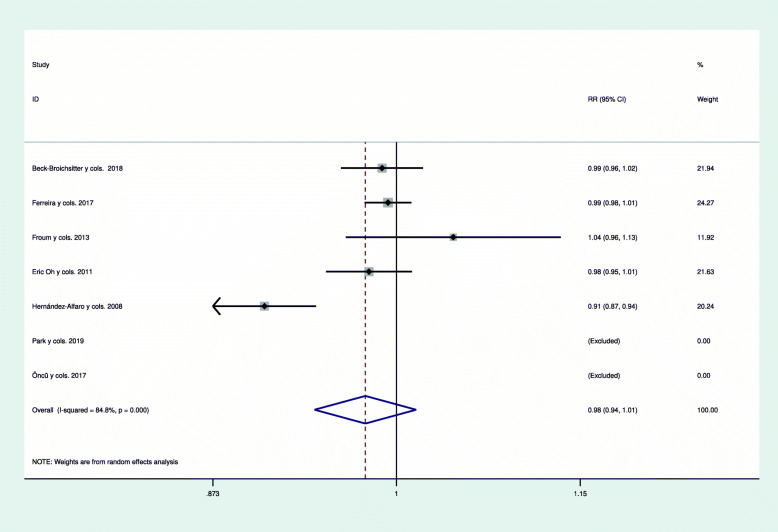
Forest plot illustrating the results in terms of implant survival rate from meta-analysis. A random effects model was used

Due to the existence of heterogeneity among the five studies included in meta-analysis, a random effects model was used to relate the survival of dental implants placed below repaired membranes and implants placed below intact membranes (*I*^2^=84.8% *p*=0.000; chi^2^=26.35 *p*=0.000). The studies by Park et al. and Öncü E et al. were not included in meta-analysis as they reported 100% survival rates for both perforated and non-perforated membranes [4, 19]. There was no statistically significant difference between the groups (*p*=0.229), with a RR of 0.977 (95% CI 0.941-1.015) (Table 7, Fig. 2).

**Table 7:** Statistical analysis of the included studies reflecting the risk ratio when comparing the implant survival rate in perforated and non-perforated membranes.

Study	RR	[95% Conf. interval]		% Weight
Beck-Broichsitter et al. [47]	0.989	0.960	1.020	21.94
Ferreira et al. [16]	0.994	0.977	1.011	24.27
Froum et al. [48]	1.043	0.963	1.129	11.92
Oh E et al. [49]	0.980	0.949	1.011	21.63
Hernández-Alfaro et al. [50]	0.907	0.873	0.942	20.24
Park et al. [4]	[Excluded]			
Öncü E et al. [19]	[Excluded]			
D+L pooled RR	0.977	0.941	1.015	100.00

Random-effects model

Heterogeneity *p* = 0.000, *I*^2^ = 84.8%

Heterogeneity chi-squared = 26.35 (d.f. = 4) *p* = 0.000

I-squared (variation in RR attributable to heterogeneity) = 84.8 estimate of between-study variance; Tau-squared = 0.0014

Test of RR= 1, *z*= 1.20 *p* = 0.229

### Publication bias

The Egger’s test (Table 8) generates a *p* value less than 0.10; this is interpreted as suspected publication bias [50]. The present systematic review obtained a *p* value of 0.739 (>0.05) indicating that small study effects did not influence the results of meta-analysis.

**Table 8:** Egger’s test

Std. eff.	Coef.	Std. err.	t	***P***>|***t***|	[95% Conf. interval]	
**Slope**	.995262	.0384472	25.89	0.000	.8729058	1.117618
**Bias**	−.9743203	2.0667276	−0.37	0.739	−9.462784	7.514143

Egger’s test for small-study effects: Regress standard normal deviate of intervention effect estimate against its standard error

Number of studies = 5, Root MSE = 2.468, Test of H0, no small-study effects, *P* = 0.739

## Discussion

Ever since Bränemark discovered osseointegration in the 1950s, numerous surgical techniques have been proposed for rehabilitating atrophic maxillae with dental implants [51]. In the absence of remaining bone in the maxillary posterior sectors, MSFA procedures may be performed with lateral window approach in order to allow implant placement either simultaneously or subsequently [52].

Maintaining the integrity of the Schneiderian membrane and sealing any perforations are critical to the success of this procedure [5, 53, 54]. Membrane perforation is a relatively frequent intraoperative event in the course of MSFA procedures with lateral window technique [17, 55] to the extent that they are considered the most frequent complication in this type of surgery (7-60%) [15, 56].

Therefore, it is essential to minimize the risk of intraoperative complications during sinus lift procedures by carrying out a preliminary study of any factors that might increase that risk including the general health of the sinus, endosseous anastomosis at the osteotomy site, lateral wall thickness, Schneider membrane thickness, residual bone crest height, the timing of subsequent implant insertion, and cortication of the sinus floor [57].

The present systematic review included total of 1162 patients who underwent 1598 lateral access MSFA procedures suffering a mean perforation rate of 30.6% (489 perforations).

The literature proposes numerous treatments to resolve these perforations. Nevertheless, although MSFA procedures are well-known and fairly commonplace, no evidenced-based guidelines for perforation closure or clear indications of when to interrupt these procedures have been established [47].

Among the treatments reported, the most widely used technique in the studies reviewed was collagen membrane repair although this was managed in different ways. While Ferreira et al. [16] placed a collagen membrane over the perforation and stabilized it with tacks to contain the graft material, Froum et al. [48] performed this repair using two separate bioabsorbable membranes.

However, collagen membranes have several drawbacks and so other therapeutic alternatives are currently being explored. In this way, De Oliveira et al. [38] assert that the resorbable membrane influences the intensity of inflammatory responses producing a reduction in bone formation, which compromises the primary stability during the placement of  the implants. Nevertheless, it should be noted that a recent systematic review of Monje et al. [58] failed to identify a statistically significant relationship between the implant mechanical (primary) stability and the implant survival rate. Similarly, Testori et al. [29] established that in the case of large perforations, the use of a collagen membrane runs a risk of displacement when the graft material is placed, so that the material is not adequately contained. Therefore, they recommend that the membranes used for the repair should cover the perforation and the surrounding area and have sufficient rigidity, even when wet, to avoid their collapse through the perforation.

A predictable two-stage approach technique to manage large perforations has been described recently in a case series by Dagba et al. [59]. These authors argue that when a large perforation occurs, further elevation of the membrane should be avoided and a collagen sponge can be folded and placed at the perforation site, which acts as a space maintainer and provides a scaffold for cell recruitment to the wounded area. The sinus augmentation procedure is then delayed by 3-6 weeks after repair of the perforation [57]. This timeframe allows the membrane to heal, facilitating re-entry [60].

Choi et al. [61] found that the use of fibrin glue for membrane repair leads to a newly formed continuous epithelium. In contrast, collagen membrane-treated perforations show extensive fibrosis, inflammatory infiltration, and an absence of epithelium [62, 63]. Öncü E et al. [19] used PRF to treat membrane perforations as this has autogenous characteristics and is an inexpensive bioactive material. Activated platelets slowly release a wide range of proteins and growth factors (BMPs, PDGFs, IGFs, VEGF, TGF-b1, TGF-b2), which act on the bone healing process and control both inflammatory response and infectious processes [64, 65]. Other authors [19, 50] have proposed suturing the membrane with resorbable material. However, in addition to the inherent difficulty of the procedure, this technique is only recommended as a single treatment in perforations of up to 5 mm due to limited access and the friability of the membrane [12, 38, 50]. Park et al. [4] observed that the simple formation of a blood clot after perforation did not lead to unfavorable clinical and radiographic results. Testori et al. [29] postulated that small perforations can be self-repairing providing the sinus membrane folds back on itself.

This systematic review showed that knowledge of the exact size of the membrane perforation is essential for deciding on the right treatment plan. Although a wide variety of treatments have been reported, a series of guidelines can be followed. Once the membrane perforation has been made, it is necessary to complete the MSFA without further enlargement of the perforation. When the procedure is terminated, the size of the perforation will determine the treatment needed and the material required.

The results of our review showed that implants inserted below repaired membranes (97.71%) had a slightly lower survival rate compared with implants inserted below intact membranes (98.88%) (RR 0.977 (95% CI 0.941-1.015). However, the difference in survival rates between perforated and non-perforated membranes was not statistically significant (*p*=0.229). Regarding these findings, we agree with Becker et al. [12] who concluded that with appropriate treatment, intraoperative sinus membrane perforations do not represent a higher risk of implant loss, infectious complications, or displacement of the graft material.

Therefore, the following treatment approaches, which correspond to those carried out in the seven articles included, were seen to obtain adequate implant survival rates.

- Perforations smaller than 5 mm can be treated by folding the membrane itself [4, 47] or with resorbable sutures [4, 50].
- When perforations are between 5 and 10 mm, the most widely recommended treatment is by means of a slow-reabsorbing collagen membrane [16, 47–50], which allows it to regenerate while facilitating closure of the communication. Adjuvant treatment may include the use of a resorbable hemostatic agent [49] or resorbable suture [16, 47] or PRF [19]. PRF activates the vascular system and promotes angiogenesis. As PRF has high strength due to its fibrin network, it can prevent graft particles from escaping into the sinus [19].
- In perforations up to 10 mm, it is thought possible to continue the MSFA procedure and even to place implants simultaneously [50].
- When perforations greater than 10 mm occur, laminar bone and a slow resorption collagen membrane should be used in combination [50]. In this case, it is advisable to place implants at a later stage [48].

Several authors [66, 67] consider that, in the case of large perforations (>10 mm), priority should be given to closing and repairing the perforation and once this has been achieved, a new osteotomy site should be prepared.

As stated above, according to the articles reviewed, implants placed adjacent to repaired perforated membranes obtained a mean survival rate of 97.68%, while those placed on intact membranes obtained (98.88%). In the systematic review and meta-analysis by Al-Dajani et al. [68], the mean survival rate of implants below membrane perforations was 93% (95% CI, 84.7-101.2), and below intact membranes 98.1% (95% CI, 93.6-102.5). Al-Moraissi et al. [15] in their systematic review observed even greater differences in implant survival between implants placed below perforated 89.65% (1022/1140) and non-perforated membranes 97.51% (3290/3374). Moreover, these authors found that there was a statistically significant association (*p*=0.06) between implant failure rate and the number of membrane perforations during MSFA procedures. Nevertheless, it should be noted that the present systematic review only included the results of MSFA procedures with lateral window approach, while Al-Moraissi et al. [15] included both, lateral and crestal approaches.

The size of the perforated membrane would appear to be the key factor influencing the implant survival rate [31, 69]. In the studies included in this systematic review, the implant failure rate increased as the size of the perforations increased (Table 5). Similarly, Hernández-Alfaro F et al. [50] also observed a lower survival rate with larger membrane perforations. Membrane perforation is also associated with a higher risk of bone graft failure and infection [70]. The use of antibiotics can help to avoid these negative consequences, promoting normal healing and the intended surgical outcomes [71].

In the present systematic review, the main complication associated with perforated membrane repair was infection. This finding concurs with Park et al. [4] who noted that a higher number of postoperative complications occurred in patients who had suffered membrane perforation during sinus lifting procedures. Similarly, Nolan et al. [72] observed that perforated sinuses presented three times the risk of bone graft failure and six times the incidence of sinusitis/infection compared with non-perforated sinuses. However, Ding et al. [73] stated that neither marginal bone loss around implants nor graft loss was affected by membrane perforation.

The present systematic review has some limitations, particularly the heterogeneity of the studies analyzed and the lack of randomized controlled clinical trials comparing different implant survival outcomes in relation to alternative strategies for managing perforated membranes. Only Beck-Broisitter et al. [47] and Hernández-Alfaro et al. [50] describe different approaches according to the size of the perforation. Therefore, further research is needed to establish a clear and validated protocol as to which form of treatment should be applied in response to different clinical scenarios.

## Conclusions

Membrane perforation is the most frequent complication during MSFA with lateral window technique. According to the findings of this systematic review, there is no statistically significant difference in subsequent implant survival rates placed below repaired membranes compared with intact membranes. Nevertheless, a higher percentage of implant failures was observed as the size of the perforations increased. The knowledge of the exact size of the membrane perforation is essential for deciding on the right treatment plan. More studies, especially prospective observational studies with longer follow-up, are needed with specific treatment guidelines and adequate sample sizes in order to provide clear and reliable results as to which form of treatment is the most effective in relation to the size of the perforation, or if some other response might be preferable.

## Supplementary Information

**Additional file 1: Annex 1.** PRISMA checklist.

## Data Availability

All data are available in the manuscript and Supplementary files.

## Abbreviations

MSFA: Maxillary sinus floor augmentation
NOS: Newcastle-Ottawa scale
RR: Risk ratio
PRF: Platelet-rich fibrin
